# From Maximization to Optimization: A Paradigm Shift in Rice Production in Thailand to Improve Overall Quality of Life of Stakeholders

**DOI:** 10.1155/2014/604291

**Published:** 2014-06-29

**Authors:** Ryoichi Doi, Supachai Pitiwut

**Affiliations:** ^1^Department of Global Agricultural Sciences, The University of Tokyo, 1-1-1 Yayoi, Bunkyo-ku, Tokyo 113-8657, Japan; ^2^Weekend Holiday Farmers' Network, 101/19-24 Moo 20, Navanakorn Industrial Estate, Khlongnueng, Khlongluang, Pathumthani 12120, Thailand

## Abstract

The concept of crop yield maximization has been widely supported. In practice, however, yield maximization does not necessarily lead to maximum socioeconomic welfare. Optimization is therefore necessary to ensure quality of life of farmers and other stakeholders. In Thailand, a rice farmers' network has adopted a promising agricultural system aimed at the optimization of rice farming. Various feasible techniques were flexibly combined. The new system offers technical strengths and minimizes certain difficulties with which the rice farmers once struggled. It has resulted in fairly good yields of up to 8.75 t ha^−1^ or yield increases of up to 57% (from 4.38 to 6.88 t ha^−1^). Under the optimization paradigm, the farmers have established diversified sustainable relationships with the paddy fields in terms of ecosystem management through their own self-motivated scientific observations. The system has resulted in good health conditions for the farmers and villagers, financial security, availability of extra time, and additional opportunities and freedom and hence in the improvement of their overall quality of life. The underlying technical and social mechanisms are discussed herein.

## 1. Introduction

Productivity is a key component of the stability of many societies. The idea of maximizing farm productivity per unit area has been supported by consumers, producers, and other stakeholders. In rice farming, an industry that is important for the global population, maximization has been attempted through various methods involving different combinations of practices and components such as irrigation regimes [1]. Some of these rice production methods, however, have involved the heavy use of chemical fertilizers, pesticides, and other products, resulting in a variety of socioeconomic and environmental problems. Many farmers have experienced financial difficulties, health issues, significant time consumption, and other problems. Meanwhile, villagers in rural areas have suffered due to polluted environments, weakened economies, and ruined communities [2]. Even consumers have often been subjected to health and financial challenges that are derived from these difficulties occurring in rural areas [3].

These negative consequences are strongly associated with heavy use of fertilizers, pesticides, labor, fuels, and other resources. The use of all these resources consumes a significant amount of the farmers' time and causes health issues among producers, villagers, and consumers. At the heart of these problems is the question of whether yield maximization should be the ultimate goal for rice producers (e.g., [4]), especially as yield maximization does not necessarily result in maximization of the total socioeconomic welfare in the production site or village, the greater municipality, the nation, and/or the world. At each agricultural production site, yield follows a saturation curve on the horizontal axis of pesticide use [5]. Therefore, when maximum crop yield is achieved, the generation of socioeconomic welfare per unit pesticide consumption is suboptimal. Similarly, yield also follows a saturation curve on the horizontal axis of fertilizer use at each site [6]. Hence the optimal amounts of pesticide, fertilizer, fuel, and other inputs in a crop production system to achieve the maximum socioeconomic welfare do not result in maximum crop yield. When higher-than-optimal amounts of these inputs are used in order to achieve maximum crop yield, the farmland is burdened with excessive pollutants such as low-molecular-weight nitrogen species (e.g., NO_3_^−^) and toxicity, while the soil is deprived of essential plant nutrients and farmers are required to contribute excessive time and labor.

To minimize these negative impacts, low-input agriculture has been heralded as a countermeasure since the 1980s. Low-input agriculture strives for the optimal use of resources [7]. Some farming systems, however, are more likely than others to achieve this form of optimization. In rice farming, the system known as system of rice intensification is one such likely low-input method [8], although it was originally meant to achieve yields significantly greater than those achieved through conventional rice farming. According to Uphoff [9], system of rice intensification generates greater yields with fewer inputs than conventional rice farming does. One unfortunate aspect of it is that its adaptation is sometimes difficult for rice farmers. The system requires intensive labor [10], a reliable water source [11], and precise knowledge [12]. System of rice intensification can, however, be successfully implemented under certain conditions such as government subsidies [13]. Unfortunately, these conditions would be hard to realize in Thailand. Furthermore, Thai society is experiencing rapid demographic changes as the population ages and migration from rural areas to cities becomes the dominant trend. Under these conditions, most rice farmers have been using heavy inputs of pesticides, fuels, chemical fertilizers, and rice seeds for direct seeding in order to maximize yield per unit area with seemingly minimum labor input, though the labor input required under this system tends to increase eventually.

As such, in this paper, we propose a new rice farming system as a countermeasure to cope with the above difficulties. The system is a set of flexible and substitutable techniques and practices. The system requires low inputs of resources, wet/dry cycles to strengthen the rice [8], techniques from biotechnology such as nitrogen fixation, wide spacing between plants achieved using a transplanter, and other feasible elements. Each farmer may choose the most suitable practices for the local site and conditions. One unique practice in the system is drying the paddy soil, especially in the tillering and rice maturity stages. The effectiveness of this practice was first recognized by a farmer in central Thailand when his irrigation system stopped working due to a technical problem at a pump station in 1970. Its discovery thus has a common starting point, namely, a shortage of irrigation water, with system or rice intensification [8]. In the last several years, the extension and adoption of the system have been significantly enhanced by local farmers' network known as the Weekend Holiday Farmers. During this period, the number of farming households using the system increased from almost zero to 5,000. The area over which the system is used has also rapidly increased over the same period from a negligible area to 16,000 ha. Its extension and adoption have been especially successful in the north and central regions of Thailand. Some farmers in other regions of Thailand have started to adopt the system, though as of April 2014 it has not yet been exported outside of Thailand.

The overall theme of the system is the optimization of rice production not yield maximization. Specifically, the system aims at the best combination of practices that results in the improvement of total quality of life for the farmers and other stakeholders. The main elements of the system are the alteration of the water management regime and the incorporation of rice straw into the soil to enrich various soil properties rather than burning it as in conventional rice farming. The adoption of the system involved on-farm experiments that were self-motivated, decentralized, evidence-based, and farmer-led [14]. Through combining the most suitable practices for each site, the system resulted in health promotion, community-level environmental recovery, financial security, generation of extra time, and other favorable effects on the farmers and the community. In this paper, we analyze and describe the ways in which the system has improved the total quality of life of farmers, rural communities, and rice consumers by clarifying the details and mechanisms involved.

## 2. Materials and Methods

### 2.1. Sites

**Figure 1:**
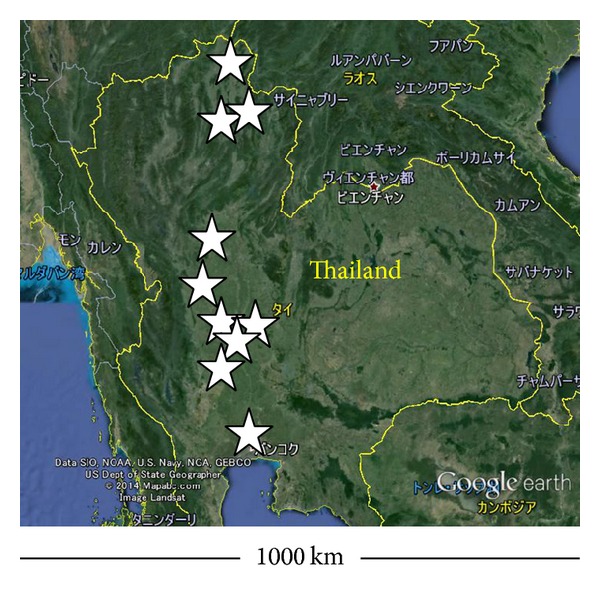
The sites at which the surveys in this study were conducted. White stars indicate the approximate locations.

In this study, paddy fields were observed at ten sites in the north and central regions of Thailand (Figure 1). At each of these sites, the system had been adopted for a length of time varying from one year (two rice seasons) to 15 years. At the northernmost site in Chiang Rai province, the mean annual temperature is 24°C and the annual precipitation is 1,734 mm. In the central region, the climate is similar to that of Bangkok, where mean annual temperature and annual precipitation are 28°C and 1,497 mm, respectively. Both regions have clear rainy and dry seasons. The climate of these regions is classified as savanna (Aw) according to Köppen [15].

### 2.2. Rice Cultivation Practices

The adoption of the system was preceded by on-farm experiments by the farmers. These on-farm experiments were not conducted in research stations by scientists like factorial experiments often are. On-farm experiments are conducted to examine, investigate, and confirm scientifically proposed hypotheses and/or scientific knowledge under the ordinary natural and socioeconomic conditions of the actual production sites [14]. The on-farm experiments in this study were made by the Thai farmers, who also incurred most of the expenses for the experiments. The on-farm experiments newly incorporated some practices that the farmers had never experienced before but that were nevertheless understandable. The new practices were introduced by outside cooperators from private sectors with scientific knowledge. Thus the process in which the experiments were followed by the adoption of the system could be seen as an evidence-based agricultural development. This development was promoted through the organization of a network of farmers, who assessed the system by comparing various factors before and after adoption. Differences between before and after the adoption were observed as effects of the adoption.

**Figure 2:**
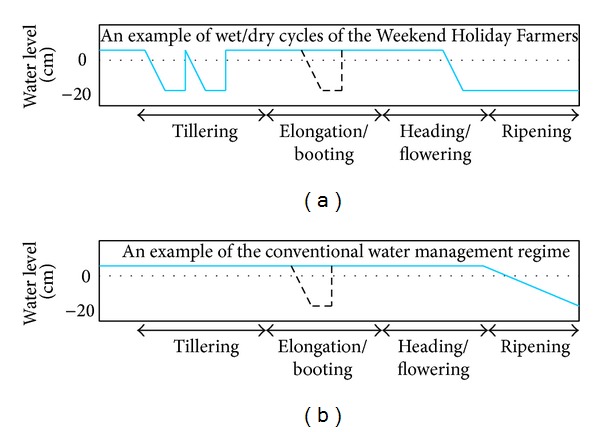
Schematic diagrams of water regimes of the Weekend Holiday Farmers' Network (a) and an example of conventional water management regime widely employed in Thailand (b) for rice production. The broken lines for the elongation/booting stage indicate optional dry periods which may occasionally be involved.

**Figure 3:**
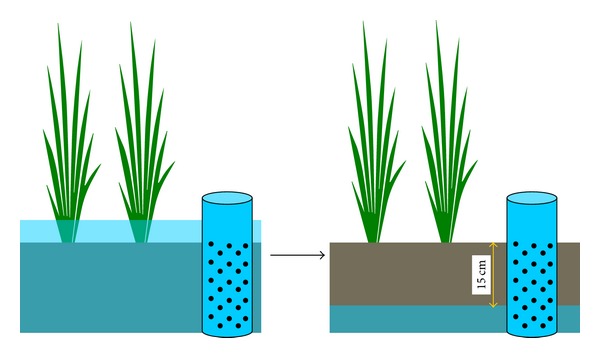
Changes in groundwater level in a paddy field in which wet/dry cycles to strengthen rice [8] are applied to enhance aeration of the soil and elongation of rice root.

Differences between the current system and the conventional rice farming system widely used in Thailand are summarized in Table 1. To describe the water regime of the Weekend Holiday Farmers' system, a schematic diagram is shown in Figure 2. The most unique aspect of this is that there are typically two periods of drying the paddy field in the tillering stage; in contrast, a large number of conventional water regimes have a single drying period around the elongation/booting period. The sinking water table can be visually observed in a 25 cm deep well prepared from plastic pipe. The water table sinks down to a depth of 15 cm from the soil surface when the soil is dried (Figure 3). Rice seedlings are planted at the age of 15 to 20 days. Seedlings are usually planted using a fuel-powered transplanter (Siam Kubota Co., Ltd., Thailand) at 5 to 10 seedlings/hill with a distance of 30 cm between hills. Organic and chemical fertilizers are often simultaneously applied from time to time depending on the response of the plant. Pesticides, especially insecticides and herbicides, are occasionally applied depending on circumstances, though their use is intended to be minimized. In this context, biocontrol agents such as fungi are employed in some cases to kill unwanted insects.* Azolla* (mosquito fern or duckweed fern) is sometimes introduced to fix gaseous nitrogen (N_2_) before the seedlings are transplanted. Introducing ducks is an alternative method of controlling weed and snail populations; their eggs can eventually be sold by the farmer as well. Manual weeders are periodically used to exclude weeds between rows of rice plants. After the harvest, the rice straw is incorporated into the soil before the next rice season. To reduce snail populations, tea seed powder [16] is sometimes applied during paddy preparation. All of these practices were presented as options, with each farmer choosing the optimal assortment of practices for his or her farm depending on his or her circumstances.

### 2.3. Interviews

Interviews with local farmers were conducted. The farmers' comments on the system were recorded, along with each farmer's demographic profile, including age, sex, and other factors. The interviews were conducted in the Thai language.

### 2.4. Paddy Field Observation and Soil Analyses

To confirm that the practices in the system actually delivered the hypothesized results, the paddy fields were carefully observed for at least a few years prior to late May 2013, when soil samples were taken. Qualitative features of the paddies such as soil surface cracking were observed and recorded as photographs. In late May 2013, soil samples were taken from the paddies using a core sampler 2.54 cm in diameter. The core sampler was inserted from the soil surface to a depth of 10 cm. At each site, four soil cores were taken and mixed in a plastic bag to make a composite soil sample representing the entire paddy field. The soil samples were air-dried, sieved at 2 mm, and analyzed. Organic matter content, water-extractable ammonium (NH_4_^+^) and nitrate (NO_3_^−^) ions, water-extractable phosphate (PO_4_^3−^), electrical conductivity, and pH were determined as previously described [17, 18]. Properties of the soils were compared with those from Tochigi prefecture, Japan. At the Tochigi site, chili peppers, rice, soybeans, and some vegetables had been cultivated in rotation. As this farming system is quite common in Japanese agricultural areas, the Tochigi soil was regarded as typical of the soil used in commercial agricultural production.

### 2.5. Statistical Analysis

The authors performed a paired *t*-test to statistically evaluate the effects of the adoption of the current system on rice yield. The SPSS 10.0.1 statistical software package (SPSS Inc.) was used to perform the *t*-test.

## 3. Results and Discussion

In their interviews, the farmers mentioned various advantages offered by the system (Table 2). One of these was that farm productivity was comparable (6 t ha^−1^ or greater) to or better (up to 8.75 t ha^−1^) than that in the central region as the most suitable region for rice farming in Thailand [19]. The farmers also commented on the minimized inputs of (1) rice seeds; (2) irrigation water; (3) chemical fertilizers; (4) pesticides; and (5) labor/time consumption. The system was found to have increased (1) the quality of the rice; (2) productivity per unit area; (3) profits and savings; (4) cooperation among villagers; and (5) total quality of life. At sites where groundwater is pumped to irrigate fields, the system also decreased electricity consumption and pump-related expenses by reducing the amount of irrigation water required. Adopting wet/dry cycles to strengthen the rice [8] is known to reduce the need for irrigation water by about 30% [20]. The mean net profit was 62,356 Baht ha^−1^ rice season^−1^ or approximately 2,000 US dollars ha^−1^ rice season^−1^. This value is fairly good for farmers who have a few to several hectares per household and multiple rice seasons each year. The greater profitability under the system was partially due to the less labor-intensive methods and the smaller expenses related to production. The system was recognized as not only profitable but also health-promoting, time-saving, and reliable, thus contributing to total quality of life, both physically and mentally. Furthermore, an easy and comfortable work environment, yield increases, and knowledge (trans-)formation were also identified as technological advantages provided by the system. The details of the farmers' responses are described here.

### 3.1. Reasons Why the System Was Adopted by the Farmers

A significant and unique aspect of the system that played a prominent role in its adoption by a large number of farmers is the method of its extension, in which the farmers played pivotal roles in the on-farm experiments and evidence-based agricultural development. Often, ideas and technologies proposed by scientists are difficult to be adopted by farmers. In addition, it is difficult for scientists to fully appreciate the importance of each production site's distinct characteristics. It is therefore helpful to have an organization that connects farmers with scientific knowledge [21]. The Weekend Holiday Farmers' Network achieves this through promoting intellectual discussions among its members and with some outside advisors from other private sectors. As it is highly decentralized, the network is free of any bureaucracy that could inhibit or even reverse progress in farming techniques [22]. Though farmers may not be familiar with certain scientific practices such as factorial experiments, it is feasible to involve scientific concepts in the farmers' efforts toward progress. Differences in various measurements taken before and after the adoption of the system show its effectiveness. As for yield (Table 2), the mean increased from 5.00 to 6.33 t ha^−1^ under the system. A paired *t*-test showed that this increase is significant (*P* = 0.020).

The farmers were self-motivated, actively adopting the system with modifications tailored to the conditions of their production sites. Because the system is a set of flexibly selectable practices, any combination that they might have chosen would be feasible and profitable. This is an advantage over top-down technology transfer, as from public/national institutes to farmers [23]. Today, the farmers in the network transmit knowledge and techniques to the other members. This farmer-farmer cooperation is often quite effective as a way to extend the system because farmers easily share and understand the realities and conditions of rice farming. These research and development activities among the farmers are already beyond participatory research and development [14, 24]. Obviously, cooperation among the members supported the farmer-led on-farm research and development as well as the connections with outside contributors from local private sectors. The farmers' responses also indicate that they have achieved evidence-based technological development because they were responsible for the results which directly determine their profits. In the future, the effects of each practice could be quantitatively determined in more detail in order to fine-tune the system.

### 3.2. Technical Aspects

As mentioned above, evidence-based research and development resulted in satisfaction of the farmers as self-motivated adopters of the system. Under conditions of optimization, the scientifically hypothesized or known effects were examined/observed under actual conditions at the rice production sites. The technical achievements enabled by the on-farm experiments and the adoption of the system will be described and discussed in this subsection.

First, because rice straw was incorporated into the paddy fields, the soils were significantly richer in soil organic matter (Table 3) compared to various other paddy soils in Thailand (2% or lower) [25]. Likewise, the pH values of the soils tended to be higher than those of most paddies in Thailand (in most cases less than 6). Often, soil pH increases when soil organic matter content increases [26]. Usually, soil organic matter is negatively charged such that it functions as a counter-ion of proton (H^+^) as a cause of acidity which drops soil pH [27]. Because of its negative charge, soil organic matter adsorbs cations such as NH_4_^+^ [28]. On the other hand, nitrate (NO_3_^−^) was proven to be adsorbed by humic acid, a major organic matter component of soil [29]. Likewise, phosphate (PO_4_^3−^, [30]) and various other ions are adsorbed by soil organic matter and released in processes of soil organic matter decomposition [27].

**Figure 4:**
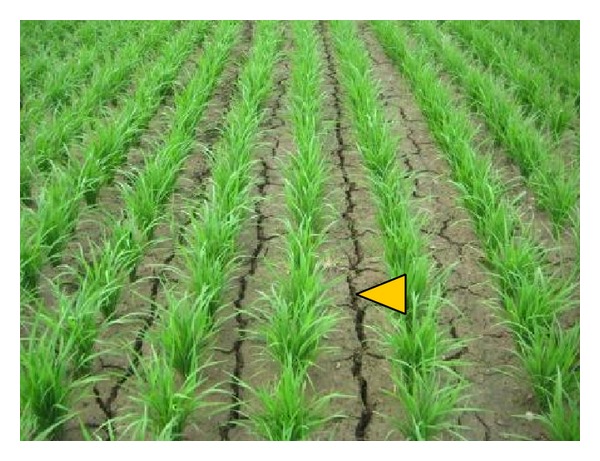
Dried soil of a paddy field in which the wet/dry cycles have been applied in the tillering stage. Note that deep cracks are recognized as lines (yellow arrowhead) between the rows of rice plants.

The adsorption and desorption processes are thought to consistently support growth of rice plants in the paddies. The paddy soils in the ten sites had low to moderate values of water-extractable low molecular nitrogen (NH_4_^+^, NO_3_^−^) and phosphate (PO_4_^3−^) ions, while those from arable fields in the Tochigi prefecture of Japan showed typical values for the ions, as in the description of various arable fields by Haney et al. [31]. Similarly, the low values of electrical conductivity for the soils of the Thai sites also indicate how effectively the plant nutrients were utilized by the rice plants (Table 3). Good rice yields have been achieved at each of the ten current sites. Thus, the low to moderate values of water-extractable low molecular nitrogen, phosphorus, and other ions in the paddy soils suggest that these ions are quite effectively supplied to the rice plants. These retained low molecular weight compounds and ions could serve as durable and versatile nutrients for rice. At the same time, their retention would help the water environment remain uncontaminated by these ions. Another advantage offered by organic matter is that organic matter-rich soils have high soil permeability and thus can quickly drain water into lower layers (Figure 4) [32]. This mechanism contributes to a firm soil surface on which people can easily walk and thus work. This effect, widely known in theory, is also visually perceivable in Figure 4.

**Figure 5:**
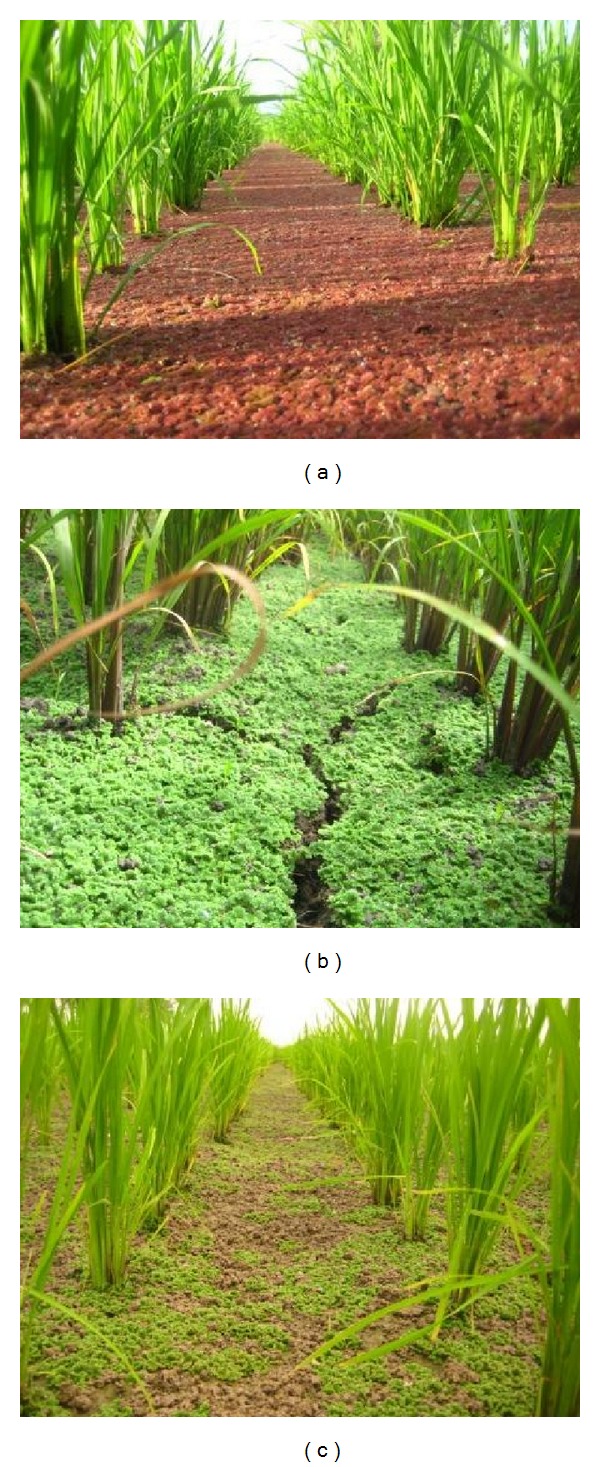
Paddy soil surface covered by* Azolla. *A reddish species in rapid propagation (a), a greenish species drying on the soil surface (b), and a greenish species decomposing on the dry soil surface (c).

The evidence-based development also confirmed another soil-amending effect of the system. Pesticide-susceptible* Azolla* was thriving in some paddies (Figure 5), suggesting that the pesticide toxicity was low [33].* Azolla* fixes nitrogen (N) at a rate of ten to more than 100 kg ha^−1^ [34]. Its ability to fix gaseous nitrogen (N_2_) in particular may significantly contribute to rice production because less than 100 kg N ha^−1^ is often adequate to achieve a good yield of 6 t ha^−1^ [35]. Therefore, the presence of* Azolla* living, dying, and decomposing on the surface of paddy soil (Figure 5) is advantageous for rice production.* Azolla* was expected to contribute to the effective use of nitrogen by rice plants (Table 3) because less excessive amounts of low molecular nitrogen compounds are necessary when* Azolla* fixes gaseous nitrogen. Furthermore, in the aerobic soil surface (Figure 4), loss of nitrogen through denitrification is suppressed. Denitrification may release gaseous nitrogen (N_2_) at a rate of up to 65 kg N ha^−1^ season^−1^ to the air in tropical climates [36]. Aerobic conditions would be favorable for minimizing the greenhouse effect by suppressing emissions of methane (CH_4_), carbon dioxide (CO_2_), and nitrous oxide (N_2_O) [37].

**Figure 6:**
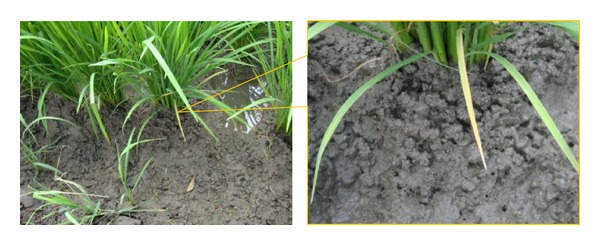
Earthworm casts visible on the soil surface of a paddy field where the current system has been applied.

In the evidence-based on-farm experiments, tea seed powder was proven to be quite effective at eliminating golden apple snails [16] due to the saponin included in the powder. At some sites, earthworms returned after the adoption of the system (Figure 6); they had formerly become extinct under conventional rice farming practices because of the toxicity of the pesticides and other chemicals used [38]. Figure 6 proves that, after the adoption of the system, the toxicity in the soil is low enough to allow earthworms to be quite active. Choosai et al. [39] observed that earthworm casts in Thai paddy soil had a variety of favorable properties for rice cultivation. Earthworms may also work with soil bacteria to oxidize toxic methane (CH_4_) to the less toxic carbon dioxide (CO_2_) [40]. These ecosystem services are certainly beneficial for roots in the paddy soil and consequently for rice production [41].

Planting rice seedlings in rows was expected to result in a reduction in rice-attacking insects. This is because the presence of a gap between rows allows direct sunlight to reach the basal part of the rice plant (Figure 4). This effect was visually confirmed. Very few leafhoppers or insects of other species were found. Under tropical climate conditions, the temperature easily exceeds 30°C when there is no surface water to cool the environment. This dry and hot environment is known to kill multiple insect species such as moths [42] and leafhoppers [43]. This works as a method of controlling insects. Use of herbicides can easily be restricted to a minimum when manual weeders are used. Manual weeding may eventually minimize or even destroy the seed bank in the paddy soil by gradually decreasing the number of persisting weed plants.* Azolla* also helps reduce weeds by covering the soil surface and competing with weed seedlings for light [34]. As another option, introducing ducks promotes and accelerates weed, snail, and insect control. The present study shows that these physicochemical and biotic practices result in fairly good perceivable effects (Tables 2 and 3, Figures 4–6), permitting the optimization of rice production by amplifying positive and minimizing negative effects on the total quality of life of the farmers and other stakeholders.

### 3.3. Contribution to the Overall Quality of Life of the Farmers and Stakeholders

Marked values of net profit ha^−1^ season^−1^ were recorded (Table 2). The profitability of farms using the system owed much to the increase in yield and the reduction in costs. There were multiple subordinate factors, however. The significant reduction in the use of pesticides and chemical fertilizers was often mentioned by the farmers. A unique result of the system was a remarkable decrease in labor intensity. In certain contexts, labor intensity can be a decisive factor in the adoption of a farming system introduced to the stakeholders [18, 44]. In the current system, labor cost and time consumption for rice production were significantly reduced. The explanations include the following: (1) decreased need for farmers to go to the paddies often because no complicated or precise water management practice is required, (2) the use of the transplanter and other machines, (3) the decreased frequency of necessary actions such as pesticide application, and (4) the dry soil surface on which walking and working are easy and comfortable. The extra time generated by the less labor-intensive system may be used for additional opportunities for the farmers. For example, some farmers were manufacturing products such as bottled fungal mycelia which would then be purchased by other farmers. As some farmers mentioned, they gained extended personal, physical, and financial freedom.

Here, an obviously decisive factor is the mechanization of the process through the use of a transplanter and other machines [19]. The Kubota transplanter is expensive for most farmers. In the sites where the system was adopted, however, the villagers were well organized to help one another despite the difficulties in organizing Thai farmers that have been reported in some circumstances (e.g., [45]). The organization/cooperation realized the mechanization and then facilitated the formerly tough work. A major reason why mechanization is important and helpful for farmers in rural regions is the aging of the population [46] in addition to human migration to cities such as Bangkok [47]. The transplanter and manual weeder are quite common among the communities/farmers. Tools that are less common but still often involved are reaper-binders, tillers, and others. Those who had not yet purchased these machines for themselves were outsourcing the practices. Some farmers were planning to buy these additional tools taking their profitability into account. They also enable the uniformity of the paddy fields, a characteristic that the farmers' organization promotes as a means of facilitating management [12].

Health-promotion and environmental protection were favorable results as in the case of the self-sufficient economy [48]. The decreased use of toxic chemicals reduced the frequency and intensity of exposure to the chemicals. The health promotion and environmental protection effects could be extended from the personal level to the community level at sites where a single farmer initially adopts the system but is later joined in it by neighboring farmers.

## 4. Conclusions

**Figure 7:**
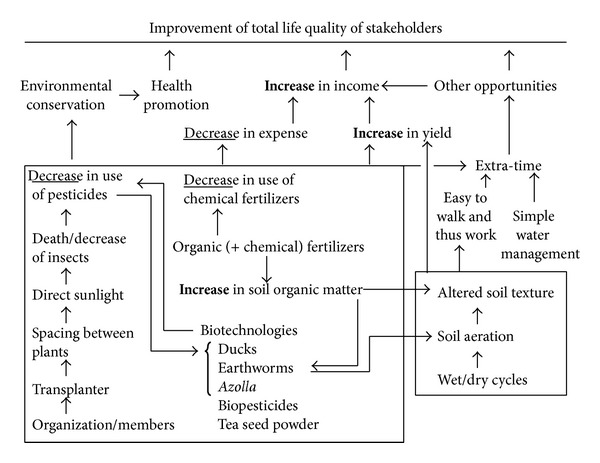
Schematic diagram of components of the Weekend Holiday Farmers' rice farming system and the effects.

The system was found to be appropriate for various rice farming sites in north and central Thailand. In addition to its profitability and the farmers' technological know-how, the system was made more feasible in Thai rural areas [49] by the existence of the Weekend Holiday Farmers' Network, which promotes intellectual cooperation among its members in addition to a few contributors from the private sector. Various hypothesized effects were confirmed in the on-farm evidence-based experiments and the subsequent adaption of the system. The system achieved increases in yield of up to 57% (from 4.38 to 6.88 t ha^−1^). This reflects the new paradigm of optimization. Optimization differs from the conventional goal of maximization of yield per unit or management of specific resources such as water [50, 51]. The crop yields observed in this study are not the maximum possible yields, but they appear sustainable in terms of all components, which can serve as determinants of the farmers' quality of life. According to the farmers' comments on the system and the results of the on-farm experiments followed by the adaption, the system has been working quite successfully so far. The adoption of the system resulted in the improvement/enhancement of environmental quality, health of farmers and villagers, financial security, freedom, and hence total quality of life (Figure 7). These effects of optimization by which various favorable outcomes are increased and negative impacts are minimized were realized due to related technical understandings which were flexibly applied by self-motivated farmers who carefully considered their site-specific conditions and alternatives.

## Figures and Tables

**Table 1:** Differences in management practice between the current rice farming system introduced by the Weekend Holiday Farmers' Network and the conventional system widely used by Thai farmers.

Practice/component	Rice farming systems	
	Weekend Holiday Farmers'	Conventional
Period of drying paddy (Figure 2)	Tillering (and elongation/booting) and ripening stages	Elongation/booting stage or no distinctive drying and before the ripening stage in which the paddy is dried
Transplanting	Mechanical transplanting with 30 cm spacing	Direct seeding
Fertilizers	Organic > chemical	Chemical *⋙* organic
Pesticides		
Insect and weed control	On demand, minimized, biopesticides (fungi etc.)	Scheduled and occasionally topped up
Snail control	Tea seed powder	Highly toxic pesticides or drainage at/after transplanting
Other biotechnologies		
Ducks	Often introduced	Seldom introduced
Earthworms and *Azolla *	Active due to low toxicity	Extinct due to toxicity

**Table 2:** Summary of interviews conducted at the sites.

Aspect	Subaspect	Value and description
Provinces		Chiang Rai, Phayao, Sukhothai, Kamphaeng Phet, Nakhon Sawan, Chainat, and Pathumthani

Age range		25–79

Cultivated area (ha)	Owned	385
	Rented	9.44

Irrigation systems		Canals, pumped groundwater

Number of seasons (year^−1^)		2 or 3

Yield	Yield for cultivar (t ha^−1^)	
	Hom Mali∗	3.13 to 5.00
	Hom Pathum	>6.25
	Phitsanulok 2	5.00 to 7.50
	Samphao Thong	6.25 to 7.50
	Suphan Buri 1	6.25
	RD 6	3.15 to 5.32
	RD 31, RD 41	5.63 to 7.50
	RD 47	5.63 to 8.75
	Yield increase after the adoption	
	Hom Mali∗	23% (4.06 to 5.00 t ha^−1^)
	Samphao Thong	57% (4.38 to 6.88 t ha^−1^)
	Suphan Buri 1	33% (4.69 to 6.25 t ha^−1^)
	RD 31	20% (6.25 to 7.50 t ha^−1^)
	RD 47	10 to 20% (5.0 to 5.5~6.0 t ha^−1^)

Cost reduction	Pesticides, insecticides, herbicides	up to 80%
	Fertilizer	up to 70%
	Others	Labor/time, water, seedlings, and seeds

Net profit (Baht ha^−1^ season^−1^)		62,356 ± 30,588 (*n* = 8)

Perceivable advantages	Quality of life	Improvement of total life quality, health promotion, greater freedom, extra time, and less anxiety (especially regarding water constraints)
	Financial aspect	Saving time saves labor and reduces investment
	Technological aspect	Easy and comfortable work, yield increase, knowledge (trans-)formation, and improved rice quality
	Social aspect	Enhanced cooperation among the villagers

*Hom Mali and the others in the subaspect column indicate rice cultivars.

**Table 3:** Properties of soils of the Thai paddy fields and the Tochigi fields in Japan.

Site		pH	Water-extractable N (mg/kg dry soil)		Water soluble phosphorus	Electrical conductivity	Soil organic matter
Country	Province or district		NH_4_-N	NO_3_-N	(mg P/Kg dry soil)	Micro- S/cm	(% dry soil-basis)
Thailand	Maesai	5.6	0.672	trace	trace	40	6.9
	Maechai	6.2	5.04	trace	trace	44	5.2
	Pong	6.2	16.5	trace	trace	50	6.0
	Kampang	6.4	1.13	trace	trace	80	5.1
	Sukhothai	6.6	4.77	trace	trace	86	5.0
	Kao Liao	6.5	1.25	trace	trace	108	9.6
	Chumsaeng	7.1	11.1	trace	trace	206	6.8
	Phayuhakiri	6.8	6.79	trace	trace	84	6.1
	Chainat	6.8	8.75	trace	trace	102	4.6
	Ladlumkaew 1	6.3	52.0	6.05	0.398	474	8.3
	Ladlumkaew 2	5.8	40.0	trace	trace	456	11.6

Japan	Tochigi, Japan 1	5.4	29.9	169	0.504	664	21.5
	Tochigi, Japan 2	6.1	9.31	55.0	0.525	750	19.1
